# Whole-brain Optical Imaging: A Powerful Tool for Precise Brain Mapping at the Mesoscopic Level

**DOI:** 10.1007/s12264-023-01112-y

**Published:** 2023-09-16

**Authors:** Tao Jiang, Hui Gong, Jing Yuan

**Affiliations:** 1 Huazhong University of Science and Technology-Suzhou Institute for Brainsmatics, Jiangsu Industrial Technology Research Institute, Suzhou, 215123 China; 2 Wuhan National Laboratory for Optoelectronics, Huazhong University of Science and Technology, Wuhan, 430074 China

**Keywords:** Whole-brain optical imaging, Optical sectioning, Micrometer resolution, Brain connectome, Neural circuits, Neuron

## Abstract

The mammalian brain is a highly complex network that consists of millions to billions of densely-interconnected neurons. Precise dissection of neural circuits at the mesoscopic level can provide important structural information for understanding the brain. Optical approaches can achieve submicron lateral resolution and achieve “optical sectioning” by a variety of means, which has the natural advantage of allowing the observation of neural circuits at the mesoscopic level. Automated whole-brain optical imaging methods based on tissue clearing or histological sectioning surpass the limitation of optical imaging depth in biological tissues and can provide delicate structural information in a large volume of tissues. Combined with various fluorescent labeling techniques, whole-brain optical imaging methods have shown great potential in the brain-wide quantitative profiling of cells, circuits, and blood vessels. In this review, we summarize the principles and implementations of various whole-brain optical imaging methods and provide some concepts regarding their future development.

## Introduction

The brain is the evolutionary pinnacle of life, controlling all aspects of the lives of humans. Numerous individuals have long hoped to decipher the mystery of how the brain works, but to date, we have yet to reveal the basic mechanisms underlying memory, thought, and consciousness. The current limited understanding of brain structure and function has resulted in a lack of effective drugs and treatments for brain disorders such as Alzheimer’s disease and Parkinson’s disease while hindering the development of brain-inspired intelligent technology. Detailed mapping of the anatomical architecture of brain cells and their brain-wide connectivity is an essential condition for elucidating how the brain works [1–4]. Efforts to “map the brain” have been ongoing for more than a century. In 1906, the Spanish neurobiologist Santiago Ramón y Cajal was awarded the Nobel Prize for his work on depicting the structure of neurons and their connections, which laid the foundation of modern neuroscience [5]. The German anatomist Korbinian Brodmann conducted a detailed study of the cortex, observing how its layers, tissues, neurons, and other cells vary in structure and size. The result was the definition of the Broadman areas, which included 52 cortical areas [6]. Based on serial histological sections, the Jülich Research Centre created a 3D probabilistic atlas of the human brain in 2020 [7], which can be further integrated with multimodal neuroimaging [8]. All these studies have extensively advanced the development of brain science.

At the macroscopic level, neuroimaging techniques such as magnetic resonance imaging (MRI), functional MRI (fMRI), and diffusion tensor imaging (DTI) have vastly increased our knowledge of the functional and structural organization of the human brain [9–12]. Nevertheless, these methods fail to capture fine structural and cellular organization due to their limited spatial resolution. At the microscopic level, electron microscopes (EMs) can provide information about structure at superresolutions of nanometers [13–15] but are limited by the low scanning speed, so 3D neuronal mapping of the whole mouse brain is not possible.

At the mesoscopic level, the lateral resolution of optical imaging methods can reach the submicron level, allowing resolution of the structure of cells, axons, and dendrites, and having the natural advantage of allowing the determination of neural circuit connectivity. Furthermore, various fluorescence labeling strategies [16–20] have greatly expanded the range of applications of optical imaging technology. However, acquiring images deep inside the brain with optical methods is challenging, as brain tissue is highly heterogeneous and strongly scatters light. In this paper, we review the technical routes and the latest progress in various whole-brain optical imaging methods.

## Challenges for Whole-brain Optical Imaging in Neural Circuit Research

The structural organization of the brain is exceptionally complex. Depending on the physical scale of interest, the brain can be divided into lobes, neural circuits, neurons, synapses, and even molecules [21]. Neurons are the basic building blocks of neural circuits; for example, the mouse brain, weighing only ~0.42 g, consists of ~70 million neurons [22–24]. The diameters of neuronal somata, arterioles, and venules are approximately tens of microns, while capillaries measure approximately several microns in diameter, and the diameters of dendrites and axon fibers measure 1 micron and below [25, 26]. However, dendrites can cover local areas of hundreds of microns, and axons can extend over long distances, sometimes spanning the whole brain. As a result, to obtain the fine structure of neural circuits, whole-brain imaging must be achieved at micron resolution, spanning several orders of magnitude [27]. This is similar to drawing a world map, which should not only cover the whole world but also accurately depict local details such as the grassroots road networks of each country.

When performing 3D optical imaging of biological tissue, the resulting dataset comprises a series of 2D images obtained by axial step scanning. Each 2D image is obtained by imaging the sample at the focal plane of the detection objective. Spatial resolution refers to the ultimate minimum structural size that a microscope can resolve. In whole-brain optical imaging, the spatial resolution includes the lateral resolution along the focal plane of the detection objective and the axial resolution along the axial direction of the detection objective. The lateral resolution is generally determined by the detection objective, calculated according to the Abbe formula *r* = *λ*/(2NA) or the Rayleigh criterion *r* = 0.61*λ*/NA [28], where *r* is the lateral resolution, NA is the numerical aperture of the detection objective, and *λ* is the imaging wavelength. The axial resolution depends on how the optical sectioning is performed and is generally worse than the lateral resolution.

The spatial resolution is usually measured by imaging standard samples such as fluorescent microspheres [29] or resolution test slides with high sampling rates. However, the resolution deteriorates in the actual imaging process due to the influence of signal intensity and density, background intensity, tissue scattering, and other factors. In addition, to fully utilize the system’s spatial resolution, the sampling interval must be at most one-half the size of the smallest resolvable feature [30]. For actual whole-brain imaging, the voxel size should be selected according to factors including the size of the research subjects, signal intensity, imaging speed, and tissue volume. Table 1 shows the recommended voxel sizes and the estimated amount of data for different research goals.

**Table 1:** Recommended voxel sizes and corresponding estimated whole-brain data sizes for different research goals

Scales of different structures in the brain	Research goal	Recommended voxel size (μm^3^)	Total amount of single-channel whole-brain raw data (16 bits/voxel)		
			Mouse [24, 31] (~ 440 mm^3^)	Marmoset [24] (~7240 mm^3^)	Rhesus macaque [32] (~91,760 mm^3^)
Axon, dendrite: ~ 1 μm Capillary: 1 μm–10 μm Soma, arteriole, venule: 10 μm–100 μm Dendritic coverage: 100 μm–1 mm Axonal length: ~1 cm Brain: 1 cm–10 cm	Fine morphology of neurons (dendritic arbors, axon terminals)	0.3 × 0.3 × 1	8.9 TB	146.3 TB	1.8 PB
	Morphology of axon projections, dendrites, somas, and capillary networks	0.5 × 0.5 × 2	1.6 TB	26.3 TB	333.8 TB
	Soma distribution and counting, vascular network imaging	2 × 2 × 3	68.3 GB	1.1 TB	13.9 TB

TB, terabyte; PB, petabyte; GB, gigabyte.

Nonhuman primates, as species much closer to humans than rodents, are irreplaceable for our understanding of cognitive functions, brain diseases, and therapies [33–36] but are also very difficult to handle and expensive, and research involving them is time-consuming. By leveraging the species’ reproductive biology and genetic engineering, nonhuman primate brain studies have become an essential part of various international brain projects [37–39]. The Japan Brain/MINDS (Brain Mapping by Integrated Neurotechnologies for Disease Studies) [40] and the China Brain Science Project [41] use marmosets and macaques as the primary research objects, respectively. As one of the smallest anthropoid primates, the marmoset brain weighs ~7.78 g and contains ~630 million neurons [23, 24, 42]. The macaque, a long-standing primate model for neuroscience research, has a brain that weighs ~87.35 g and contains ~6.37 billion neurons [23, 42]. Due to their enormous sizes, optical imaging of the whole brains of nonhuman primates is far more difficult than that of mouse brains.

## Technical Routes for Achieving Whole-brain Optical Imaging

**Fig. 1:**
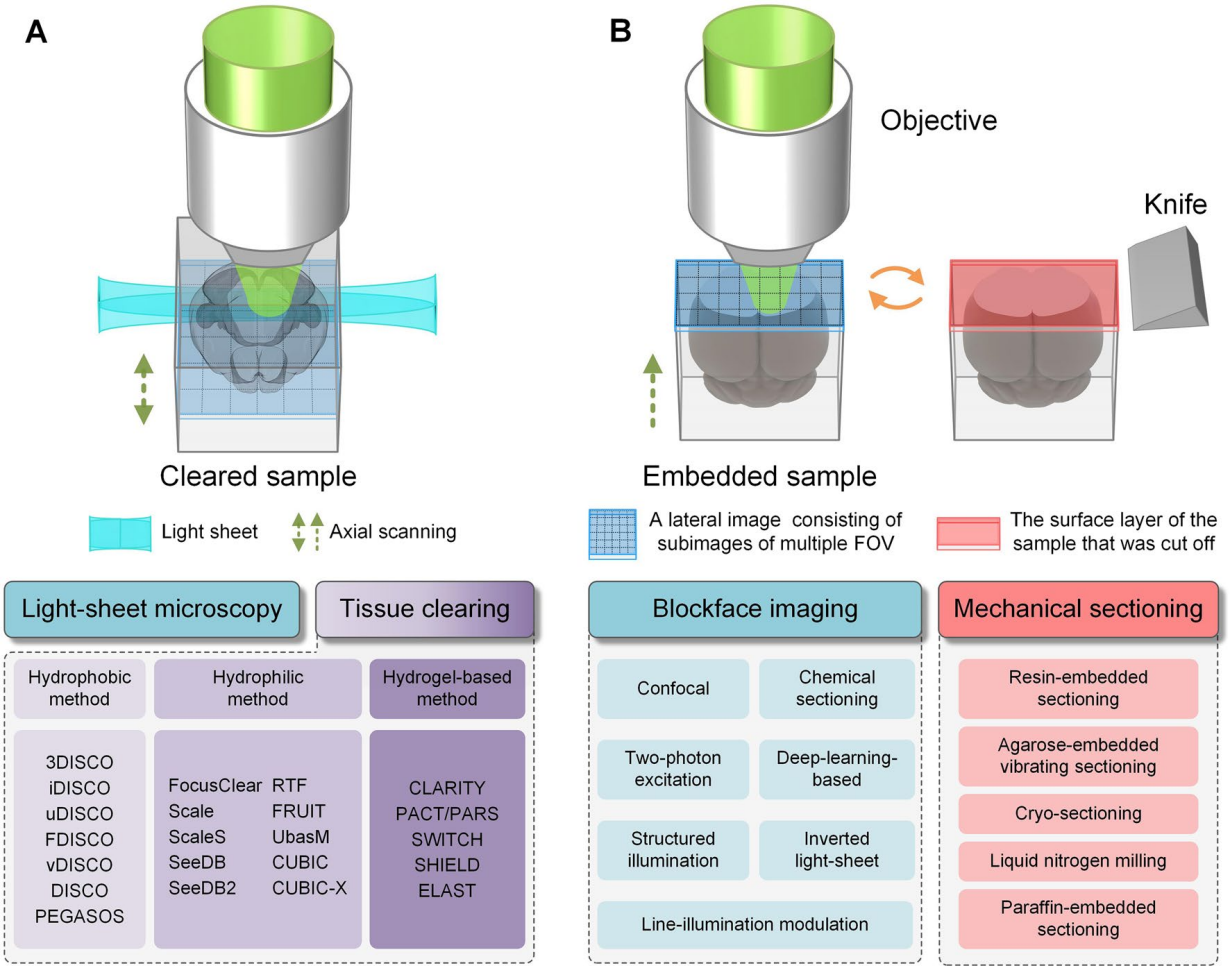
Two technical routes for achieving whole-brain optical imaging. **A** Schematic of tissue clearing combined with light-sheet microscopy for whole-brain optical imaging. **B** Schematic of block-face imaging combined with mechanical sectioning for whole-brain optical imaging.

Due to the scattering and absorption of light by biological tissues, classical optical methods such as confocal and two-photon microscopy have limited imaging depth, reaching only tens to hundreds of microns into the mouse brain [43, 44]. To obtain micron-resolution 3D datasets of centimeter-sized samples, whole-brain optical imaging must solve the following two problems. First, the out-of-focus signal must be suppressed to obtain a single high-resolution 2D image, which can be achieved by various optical sectioning methods such as confocal, two-photon excitation, structured illumination, and light sheet sectioning [43, 45–48]. The second is to surpass the limitation of optical imaging depth to axially scan large samples. The different existing whole-brain optical imaging techniques can be divided into two technical routes according to the way they overcome the optical imaging depth limit: tissue clearing-based techniques and histological sectioning-based techniques (Fig. 1).

Tissue clearing, a century-old approach [49], refers to a collection of techniques that render biological samples transparent by following the simple rule of refractive index matching to eliminate scattering [50]. However, in practice, the choice of tissue-clearing reagents and design protocols requires consideration of many factors, including molecules of interest, maintenance of sample structures, endogenous fluorescent proteins, and immunolabeling [51]. The tissue-clearing workflows are too numerous and complicated to categorize accurately, but they can be broadly divided into the following modules: fixation, pre-treatment, delipidation, labeling, and refractive index matching [51]. According to the type of reagent used, tissue-clearing approaches can be divided into hydrophobic, hydrophilic, and hydrogel-based methods [18, 52, 53]. Hydrophobic methods, also called solvent-based clearing methods, use organic solvents to achieve fast and complete transparency of the intact sample. However, some hydrophobic techniques can quench the signal of a fluorescent protein. Representative hydrophobic methods include 3D imaging of solvent-cleared organs (3DISCO) [54], immunolabeling-enabled 3DISCO (iDISCO) [55], ultimate 3DISCO (uDISCO) [56], DISCO with superior fluorescence preserving capability (FDISCO) [57], nanobody(V_H_H)-boosted 3DISCO (vDISCO) [58], stabilized 3DISCO (sDISCO) [59], and polyethylene glycol (PEG)-associated solvent system (PEGASOS) [60]. Hydrophilic methods using water-soluble reagents cannot achieve complete transparency like hydrophobic methods but have better biocompatibility and biosafety with brain tissues. Representative hydrophilic tissue-clearing approaches include FocusClear [61], Scale [62], ScaleS [63], See Deep Brain (SeeDB) [64], See Deep Brain 2 (SeeDB2) [65], rapid clearing method based on Triethanolamine and Formamide (RTF) [66], FRUIT [67], Urea-Based Amino-sugar Mixture (UbasM) [68], Clear, Unobstructed Brain/Body Imaging Cocktails and Computational analysis (CUBIC) [69–72] and CUBIC-X [73, 74]. Based on the principle of securing biomolecules *in situ* through covalent crosslinking, hydrogel-based methods convert tissue into synthetic gels using polyacrylamide or into reinforced tissue gels using polyepoxide, followed by delipidation and refractive index matching. Hydrogel-based methods can achieve a good transparency effect, but some technologies require a long incubation time, and the operation protocols are complicated. Representative technologies include CLARITY [75], passive CLARITY technique/perfusion-assisted agent release *in situ* (PACT/PARS) [76, 77], system-wide control of interaction time and kinetics of chemicals (SWITCH) [78], stabilization to harsh conditions *via* intramolecular epoxide linkages to prevent degradation (SHIELD) [79], and entangled link-augmented stretchable tissue-hydrogel (ELAST) [80].

Once the tissues are rendered transparent, light-sheet microscopy is the preferred choice for rapid subcellular-resolved volumetric imaging of intact samples. Different from the conventional microscope, the illumination and detection path are separated in a light-sheet microscope [48, 81]. A thin light sheet illuminates the sample from the side to achieve selective fluorescence excitation. The optical axis of the detection arm is perpendicular to the light sheet, and the focal plane of the objective coincides with the light sheet. Except for the time required to move the field of view (FOV), the imaging speed of light-sheet microscopy is limited only by the acquisition rate of the camera. Light-sheet illumination effectively reduces photobleaching and phototoxic effects. The thickness of the light sheet and the NA of the detection objective together determines the axial resolution of light-sheet microscopy. However, to cover the imaging range of the whole mouse brain, thick illumination light sheets and long working-distance, low-NA, large field-of-view detection objectives are generally needed [50, 51, 82].

Another technical route for whole-brain imaging is the combination of block-face imaging with histological sectioning: the restriction of optical imaging depth is overcome by alternating the imaging and sectioning processes. Various optical sectioning methods, including confocal, two-photon excitation, structured illumination, and inverted setup light-sheet microscopy [48], as well as other emerging techniques (chemical sectioning [83], deep learning-based optical sectioning [84], and line-illumination modulation [85]), can be used to obtain images of shallow parts of the sample. Moreover, diverse histological sectioning techniques have been developed for embedding and sectioning the whole mouse brain. For example, resin-embedded sectioning can achieve submicron accuracy [86, 87], agarose-embedded vibrating sectioning can preserve the morphology of the tissue well [88–90], cryosectioning or liquid nitrogen milling can maintain the biochemical characteristics of the sample [91, 92], and paraffin-embedded sectioning enables the semithin sectioning of large tissues [93, 94]. The imaging quality and speed of such whole-brain imaging methods largely depend on the selection and implementation of the imaging and sectioning techniques. Due to the time-consuming nature of the tissue sectioning, the imaging speed of such methods is lower than that of tissue-clearing-based, whole-brain imaging methods. Thankfully, however, the sample preparation is simple with this technical route, and higher resolutions and more uniform data quality can be more easily achieved.

## Light-sheet Microscopy for Whole-brain Optical Imaging

The concept of perpendicular illumination was first proposed >100 years ago [95] in the observation of colloidal particles. In 2004, Huisken *et al*. developed selective plane illumination microscopy (SPIM) to generate 3D images of millimeter-sized transparent medaka embryos [96], achieving the first application of light-sheet microscopy in developmental biology. In 2007, Dodt *et al*. proposed ultramicroscopy combined with a hydrophobic tissue-clearing solution and thus were the first to use light-sheet imaging on artificially transparent mouse brains [97]. Over the past several decades, various light-sheet microscopy techniques have been proposed and have rapidly been applied in biological imaging [48, 81, 98, 99].

**Fig. 2:**
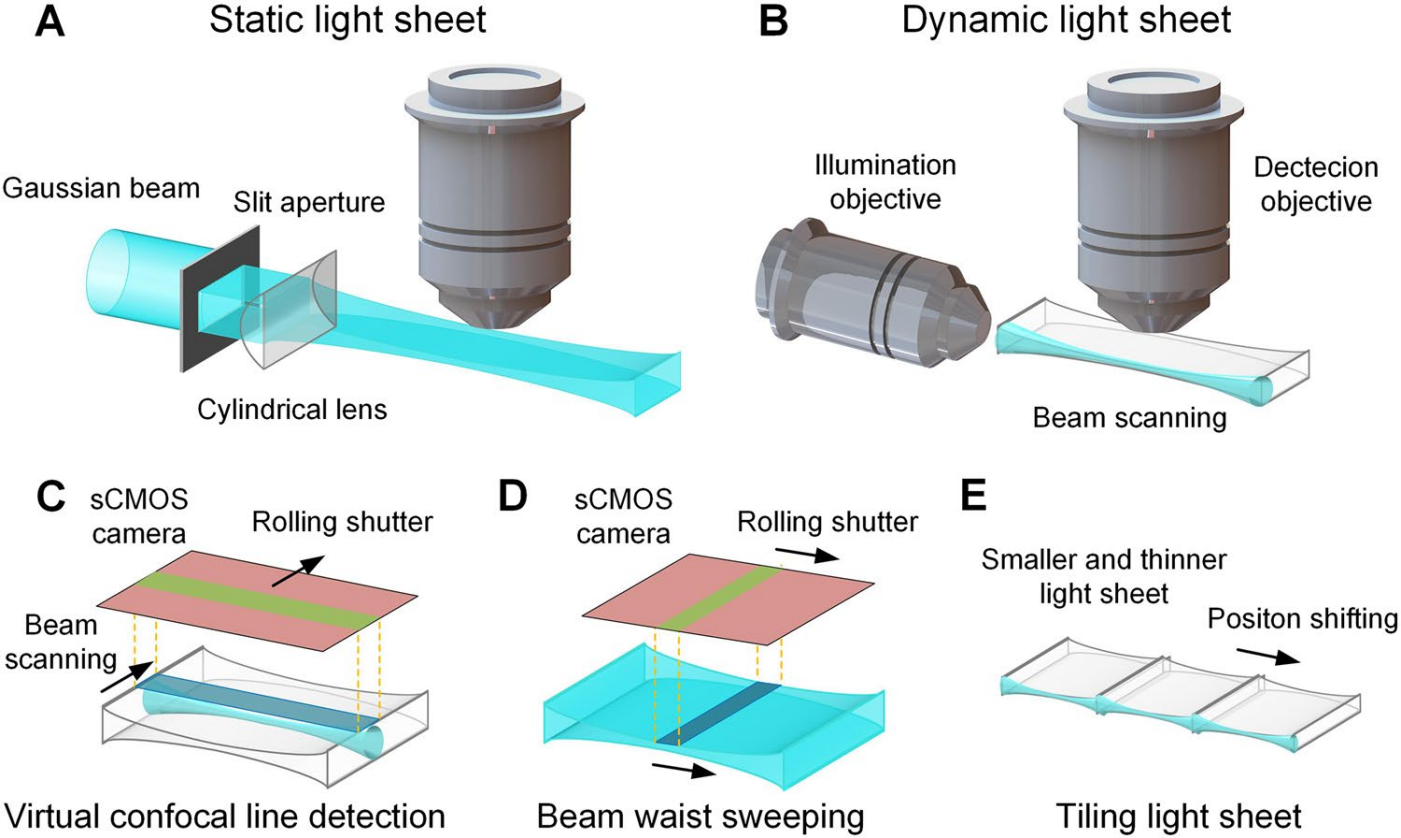
Schematic of the principle of different light-sheet microscopes. **A** A static light sheet is formed by focusing a Gaussian beam with a cylindrical lens. **B** A dynamic light sheet is generated by scanning a laser beam. **C** Virtual confocal line detection is performed by synchronizing the rolling shutter of an sCMOS (scientific complementary metal-oxide-semiconductor) camera with illumination beam scanning. **D** The axial resolution can be improved by synchronizing the rolling shutter of an sCMOS camera with beam waist sweeping. **E** The axial resolution can also be improved by tiling smaller and thinner light sheets.

The generation of light sheets is a crucial part of implementing light-sheet microscopy. An ideal light sheet has a large lateral extent with a uniformly small axial extent to achieve uniform and thin light-sheet illumination and fluorescence excitation in a wide FOV. However, limited by physical principles and optics, this ideal light sheet is difficult to achieve. Light sheets are typically divided into two categories; static light sheets formed by focusing a Gaussian beam with a cylindrical lens [96] (Fig. 2A) and dynamic light sheets generated by scanning a laser beam across the FOV [100] (Fig. 2B). Non-Gaussian beams such as Bessel beams [101, 102], Airy beams [103], and lattice-based light sheets [104] can be used in dynamic light-sheet implementation to improve the axial resolution of high-speed, volumetric functional imaging. However, Gaussian beams are most commonly used to produce light sheets in practice for imaging large, cleared neural tissue. The optical sectioning resolution of a light-sheet microscope is derived from the product of the illumination and detection point spread functions (PSFs) [48, 81]. For Gaussian beams, a higher-NA illumination objective produces a thinner light sheet and higher axial resolution but with a shorter Rayleigh range and a smaller FOV. In addition, using a high-NA detection objective can increase both the lateral and axial resolution. However, the detection objective NA is limited by geometric and immersion medium constraints and the working distance [81, 105]. Additional practical engineering problems include the inhomogeneity in the depth of the cleared sample and the refractive index mismatch between the sample, solution, air, and container. These trade-offs make it challenging to achieve high-resolution optical sectioning with a large FOV and thus a uniform spatial resolution and image quality over an intact sample of centimeter size.

Ultramicroscopy [97] uses a cylindrical lens to generate a Gaussian static light sheet and two opposing illumination arms to illuminate the sample from both sides. This method has been shown to achieve complete imaging of the excised hippocampus and whole embryonic mouse brain, with an axial resolution of several microns. Saghafi *et al*. [106] achieved a more uniform light sheet with a longer Rayleigh range using aspheric optics, further improving the imaging quality of ultramicroscopy. Digital scanned laser light-sheet fluorescence microscopy (DSLM) [100], developed by Keller *et al*. in 2008, was the first technique capable of generating dynamic light sheets by rapidly scanning a micrometer-thin laser beam and was used for the *in vivo* imaging of zebrafish embryos. This approach improved illumination uniformity, efficiency, and image quality over cylindrical lens-based light-sheet generation schemes. Mertz *et al*. used an acousto-optic modulator (AOM) to achieve structured illumination during beam scanning. They demonstrated the ability to suppress out-of-focus backgrounds through partial imaging of a cleared mouse brain [107]. The confocal light-sheet microscopy (CLSM) technique [108] proposed by Silvestri *et al*. used confocal detection to reject background signals but required sophisticated synchronous scanning. Baumgart *et al*. realized virtual confocal line detection conveniently by synchronizing the rolling shutter of a scientific complementary metal-oxide-semiconductor (sCMOS) camera with the scanning illumination beam [109] (Fig. 2C). The CLARITY-optimized light-sheet microscope (COLM) [110] was designed with double-sided beam scanning illumination and virtual confocal line detection. It can acquire an entire clarified mouse brain dataset in ~4 h with a 10×, 0.6-NA detection objective. Spherical-aberration-assisted extended depth-of-field (SPED) light sheet microscopy [111] extends the field depth by subtly inducing spherical aberrations, enabling high-speed volumetric imaging without needing to scan the detection objective. This technique is capable of rapid subcellular resolution imaging of 1-mm-thick CLARITY mouse brain samples and cellular resolution Ca^2+^ imaging of entire zebrafish nervous systems.

To overcome the disadvantage of the uneven axial thickness of Gaussian beam-based light sheets and further improve the axial resolution in a large FOV, a higher-NA beam waist can be swept along the sheet’s propagation direction, as proposed in axially swept light-sheet microscopy (ASLM) [112] (Fig. 2D). Synchronizing the sweeping with a rolling shutter allows large-volume imaging of cleared tissues with consistently high axial resolution [113–116]. However, this method sacrifices some integration time benefits and the original selective excitation advantage of light-sheet illumination. Among these methods, mesoscale-selective plane-illumination microscopy (mesoSPIM) [114] was designed as an open-hardware project for building and operating a modular light-sheet microscope that utilized an electrically tuneable lens (ETL) for excitation beam waist sweeping. Cleared-tissue axially swept light-sheet microscopy (ctASLM) [115] achieved isotropic high-resolution imaging over large FOVs through fast aberration-free remote focusing. This technique acquired a millimeter-sized cleared mouse bone marrow dataset with an isotropic resolution of ~300 nm. Unlike sweeping the illumination beam waist, a higher axial resolution within a large FOV can also be achieved by tiling a smaller but thinner light sheet [117] (Fig. 2E). Tiling light-sheet microscopy (tiling LSM) [118] uses this approach to achieve fast imaging of transparent tissues at multiple resolution scales. A multi-immersion open-top light-sheet microscope [119] uses a configuration with the illumination and collection objectives at 45° to the vertical axis, and they are placed below (rather than above) the cleared specimen. This approach increases the ease of use and throughput and enables simple mounting of multiple samples processed with various clearing protocols. The further upgraded hybrid open-top light-sheet microscopy (hybrid OTLS) [120] can achieve versatile multiscale volumetric imaging >12 cm × 7.5 cm × 1 cm by combining a unique non-orthogonal dual-objective and conventional (orthogonal) open-top light-sheet architecture. This flexible system provides an efficient solution for different imaging requirements in terms of resolution, sample size, and tissue-clearing protocol. In addition, light-sheet microscopy can perform multiview imaging of the same sample [121, 122] and achieve improved spatial resolution and image contrast by implementing multiview deconvolution and other algorithms [123, 124]. The multiangle-resolved subvoxel selective plane illumination microscope (Mars-SPIM) [125] first acquired low-resolution raw datasets of the cleared whole mouse brain from 8 views in 30 min with a Gaussian static light-sheet microscope and reconstructed a digital atlas with ~1 µm^3^ isotropic voxels. Content-aware compressed-sensing (CACS) light-sheet microscopy [126] images the cleared mouse brain at a low resolution under two opposite views in ~10 min with a dual-side confocally-scanned Bessel light-sheet microscope and then restores the isotropic voxel resolution to 0.5 μm^3^ and improves the signal-to-noise ratio for the two-view fusion 3D image. This type of computational method to improve the resolution requires at least several hours of additional post-computation time. More importantly, the low NA used in the acquisition of original low-resolution data enables the system to obtain a large field of view and accelerate the imaging speed, but also greatly limits the detection of fine structures with weak signals. Therefore, the digitally-reconstructed high-resolution data from the original data acquired at a low NA are not comparable to those obtained directly from optical imaging with a high NA.

As an increasing number of commercial products (LaVision, Zeiss, Olympus, Leica, Nuohai, Applied Scientific Instrumentation, and Bruker) become available, light-sheet microscopes are increasingly becoming routine equipment in microscope facilities. While light-sheet imaging allows for high-throughput volumetric data acquisition, clearing methods must make the sample highly transparent to avoid excessive residual scattering and absorption for whole-brain imaging. Light-sheet microscopy generally enables rapid imaging of centimeter-sized cleared mouse brains in hours at micron-level resolution and fine imaging of local areas at submicron resolution. Consequently, this approach is an attractive strategy for quantitative brain-wide cell profiling.

## Whole-brain Optical Imaging Based on Block-face Imaging and Histological Sectioning

In contrast to tissue-clearing strategies, the optical imaging depth can also be extended by combining block-face imaging with histological sectioning. This automated block-face whole-brain imaging approach combines top-view optical sectioning imaging with integrated tissue sectioning; it can be viewed as an automated high-throughput implementation of the traditional, manual histological procedure [127, 128]. Although this method results in a prolonged sectioning time, the sample embedding process is simple, and it is easier to achieve uniform high resolution and image quality for large tissue samples. This advantage is essential for observing delicate structures such as axons and dendrites that are distributed brain-wide and measure only 1 micron or less in diameter. Representative approaches include serial two-photon tomography (STP), block-face serial microscopy tomography (FAST), and micro-optical sectioning tomography (MOST) series of technologies.

## Serial Two-photon Tomography

**Fig. 3:**
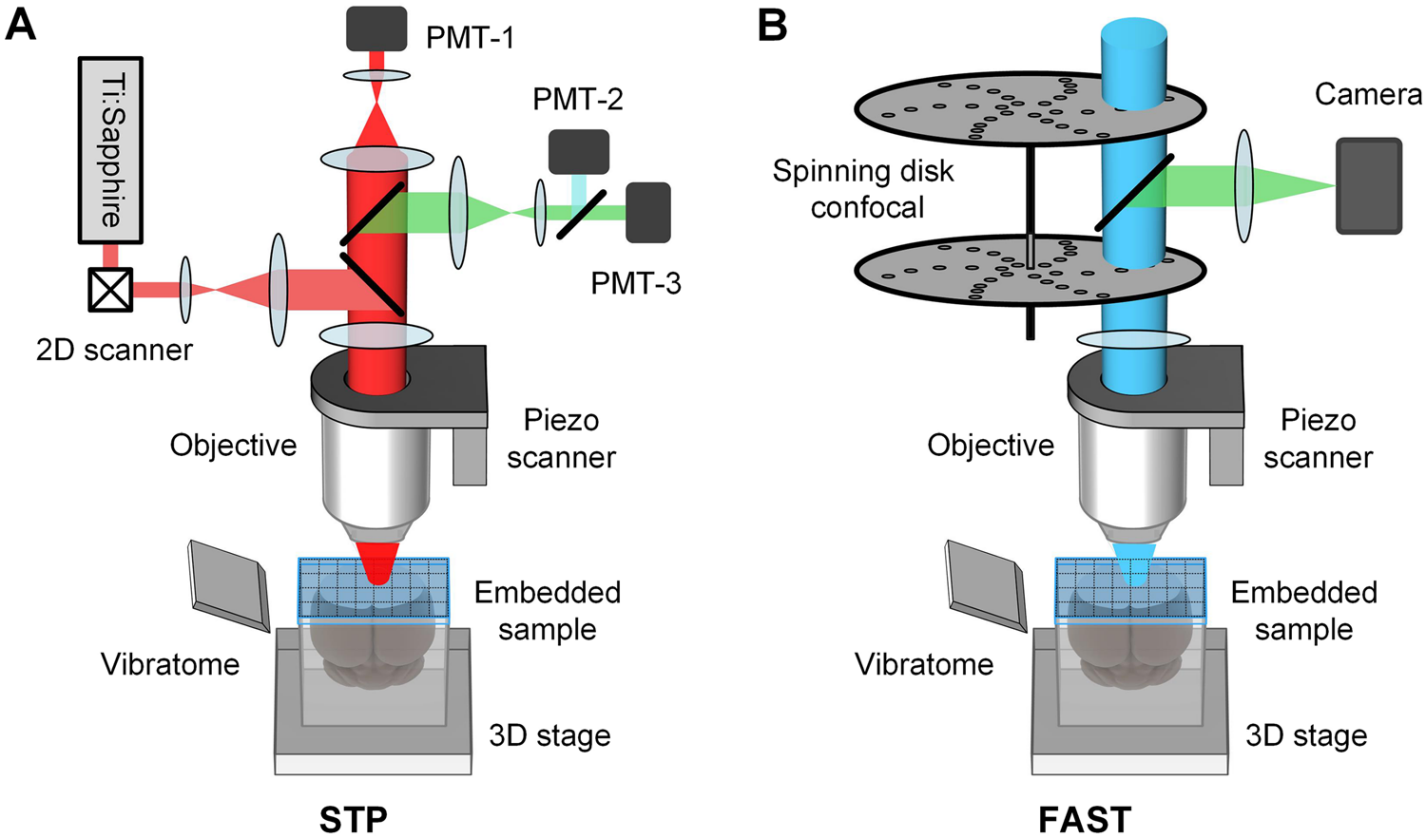
Schematic of the serial two-photon tomography (STP) and block-face serial microscopy tomography (FAST) systems. PMT, photomultiplier tube. The picture is redrawn according to the original system schematic with permission from [88, 136].

In 2007, two-photon tissue cytometry was developed by Ragan *et al*. [129], combining two-photon microscopy with paraffin-embedded sectioning to enable 3D imaging of intact mouse hearts at a voxel size of 0.78 μm × 0.78 μm × 2 μm. In 2012, an improved STP technique was developed that employs a two-photon microscope based on high-speed galvanometric scanners and vibrating sectioning of agarose-embedded brains with minimal detrimental effects on the fluorescence and sample morphology [88] (Fig. 3A). The imaging plane is located 50 μm below the surface to obtain undisturbed optical images, and lateral 2D images of the entire brain coronal sections can be imaged as a mosaic of FOVs. This technique has high lateral sampling rates (2 μm, 1 μm, and 0.5 μm optional); however, due to the limited imaging throughput, the axial sampling interval is typically set to 50 μm, making it impossible to track continuous signals in 3D. In 2016, Economo *et al*. [130] used a resonant scanning galvanometer and high excitation power to further improve the STP technique in high-speed volumetric STP tomography. They devised a low viscosity clearing solution that enabled reliable vibrating sectioning of gelatin-embedded brain samples over many days and allowed an imaging depth >200 μm. With this platform, a mouse brain dataset with a voxel size of 0.3 μm × 0.3 μm × 1 μm could be obtained in 8 days–10 days, enabling the visualization and reconstruction of long-range axonal arbors. However, additional data registration was required due to the inevitable tissue deformation introduced by the sectioning of cleared samples, and the imaging speed of this point-scan method could not be improved further without much difficulty. The MouseLight project used this platform to reconstruct >1000 projection neurons in the motor cortex, thalamus, subiculum, and hypothalamus [131].

STP uses two-photon excitation to achieve high-quality optical sectioning; however, point-by-point scanning significantly limits throughput. Therefore, a more typical application of STP is whole-brain imaging with an axial sampling interval of 50 μm–100 μm in 6.5 h–21 h. Due to the simple and deformable-free agarose embedding, STP was used at the mesoscale to generate a series of two-dimensional images spanning all regions of the brain, enabling brain-wide quantitative cell profiling [132–134] and region-to-region connectivity (The Allen Mouse Brain Connectivity Atlas) [135].

## Block-face Serial Microscopy Tomography

Similar to STP, the FAST technique proposed by Seiriki *et al*. in 2017 also uses vibrating sectioning to achieve 50 μm–80 μm slicing of agarose-embedded samples. The difference is that FAST also uses a spinning disk-based confocal microscope to optically section at depths of up to 100 μm below the surface and monochromatically image a whole mouse brain in 2.4 h with a voxel size of 0.7 μm × 0.7 μm × 5 μm [136, 137] (Fig. 3B). This method employs a Nipkow spinning disk, which projects highly parallelized excitation light beams to achieve high imaging rates and has optical sectioning capabilities comparable to those of traditional confocal microscopy. FAST has also been used to image the marmoset brain at subcellular resolution, but the low axial sampling rate limits the continuous tracing of axons and dendrites.

The sparse imaging and reconstruction tomography (SMART) system, proposed by Chen *et al*. in 2021, also uses a spinning-disk confocal system and vibration sectioning configuration but is equipped with a high-NA objective to achieve a voxel size of 0.3 μm × 0.3 μm × 1 μm and can be combined with tissue clearing to increase the imaging depth to 250 μm [138]. This technology adopts a sparse imaging strategy that combines real-time data analysis with instrument control. Under the assumption that the labeled individual neurons are structurally continuous, the strategy first acquires a single slice image to determine the area where the signal is located, followed by further volume imaging. As a result, a whole mouse brain dataset with sparsely labeled neurons is acquired in ~20 h. However, this approach inherently possesses a fixed maximum imaging speed, can lead to data loss due to the missed detection of signals, and is unsuitable for samples with widely distributed signals.

## Micro-optical Sectioning Tomography

**Fig. 4:**
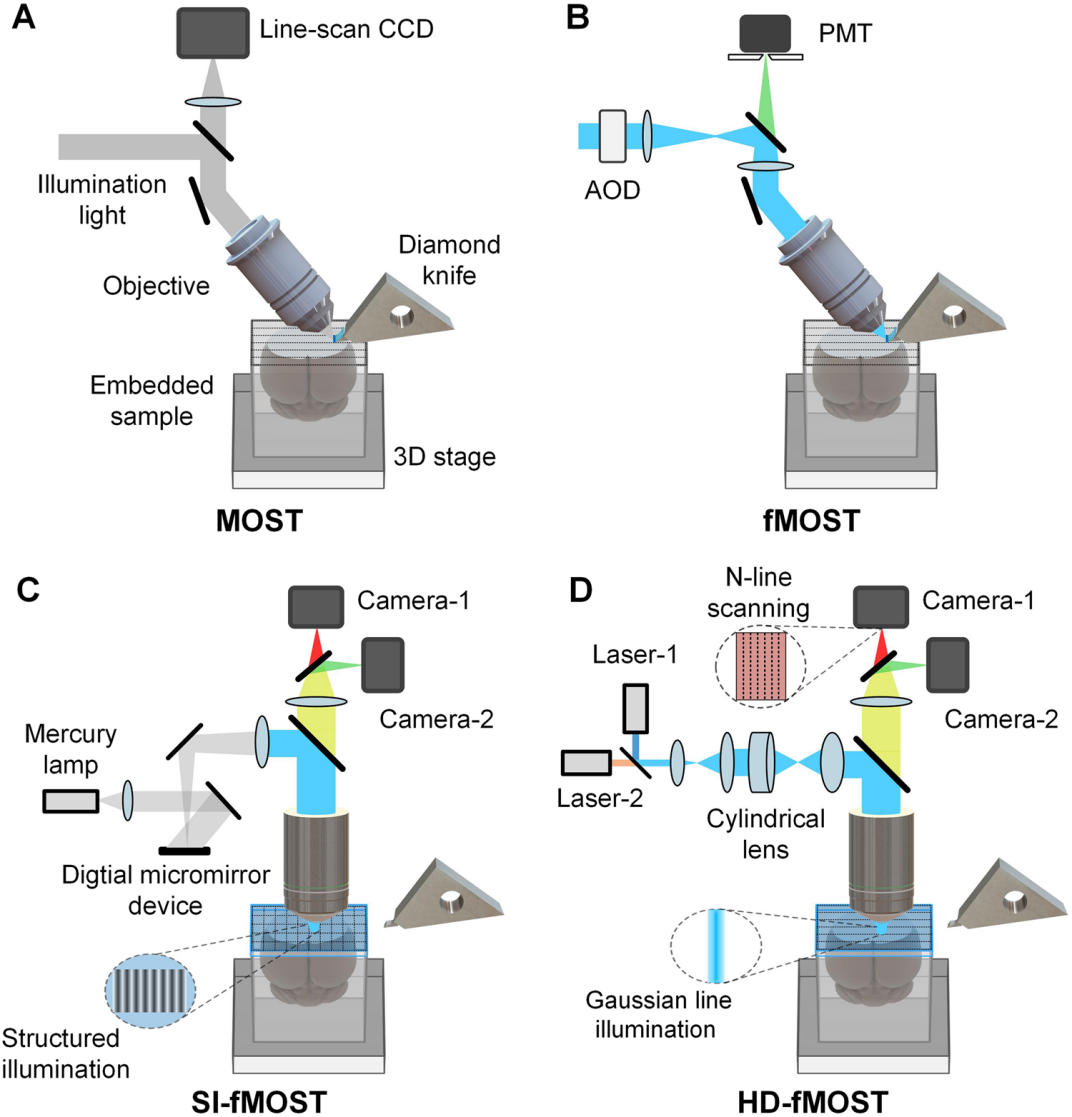
Schematic of the MOST, fMOST, SI-fMOST, and HD-fMOST systems. CCD, charge-coupled device. PMT, photomultiplier tube. AOD, acousto-optic deflector. The picture is redrawn according to the original system schematic with permission from [85, 86, 142, 145].

In 2010, Li *et al*. proposed a MOST system based on automatic precision sectioning and line-scanning imaging. MOST uses a diamond knife to continuously cut resin-embedded mouse brain samples into 1-μm thick sections and then performs line-scanning imaging at the moment of section generation [86] (Fig. 4A). The section thickness directly determines the axial resolution of this method. For the first time, imaging of a Golgi-stained whole mouse brain [139] with a voxel size of 0.33 μm × 0.33 μm × 1 μm was achieved in 242 h, with a total of 15,380 coronal sections. Using this system to image Nissl-stained samples, the cellular and vascular configurations in the whole mouse brain can be visualized at a submicron voxel resolution with high image quality [140, 141]. In 2013, fluorescence micro-optical sectioning tomography (fMOST) was developed, improving the imaging part of MOST using an acousto-optic deflector (AOD) to achieve long-term stable confocal laser scanning imaging [142] (Fig. 4B). Combined with a resin-embedding method that preserves the fluorescent protein signal [143], fMOST can image a fluorescence-labeled, whole mouse brain with a voxel size of 1.0 μm × 0.8 μm × 1.0 μm in 447 h, which demonstrated for the first time the tracking results of the uninterrupted long-range axon projection of a single neuron across the whole brain [27].

To further improve the imaging speed and system robustness, structured illumination fluorescence micro-optical sectioning tomography (SI-fMOST) was developed by adopting a high-throughput structured illumination microscope [144] to perform block-face imaging of the entire sample section in a mosaic manner (Fig. 4C). Due to the submicron precision of resin-embedded sectioning, the system can implement real-time counterstaining of the sample surface in the whole-brain imaging process. Compared to the simultaneous slicing and imaging approach adopted by MOST and fMOST, the block face-based SI-fMOST decouples the imaging from the sectioning, further enhancing reliability and reducing distortions and data loss. As a result, a colocalized whole-brain dataset of both fluorescence-labeled neurons and counterstained cell bodies with a voxel size of 0.32 μm × 0.32 μm × 2 μm was acquired in 3 days [145], significantly facilitating the precise tracing of long-range projections and accurate location of nuclei. Chemical section fluorescence micro-optical sectioning tomography (CS-fMOST) adopts a unique chemical reactivation method [83] to illuminate only the top, submicron-thick layer of the sample for imaging without background fluorescence. This chemical reactivation method is achieved by chemically switching the fluorescent state of the labeled proteins off and on. Therefore, the imaging module does not have to implement the optical sectioning function, dramatically reducing the system’s complexity. CS-fMOST also employs a high-throughput time-delay integration (TDI)-based line-scanning widefield microscope [146], allowing multicolor whole-brain imaging with a voxel size of 0.23 μm × 0.23 μm × 1 μm within 6 days [147]. High-definition fluorescence micro-optical sectioning tomography (HD-fMOST) implements a line-illumination modulation (LiMo) technique, which uses the natural intensity modulation of Gaussian line illumination to achieve high-throughput line scanning with remarkable background inhibition (Fig. 4D). Benefitting from LiMo, HD-fMOST can achieve high-definition whole-brain optical imaging with a voxel size of 0.32 μm × 0.32 μm × 1 μm in 111 h [85] and perform high-efficiency online data compression and processing. HD-fMOST pushes further into the limits of optical sectioning and demonstrates the potential to facilitate large-scale acquisition and analysis of whole-brain high-resolution datasets.

In addition, a variety of MOST techniques based on other imaging and sectioning methods have been developed, including two-photon fMOST (2p-fMOST), which uses two-photon excitation [148], dual-mode MOST (dMOST), for the simultaneous acquisition of Golgi-stained neurons and cytoarchitecture [149], rapid whole-brain optical tomography, capable of automatic slice collection [89], the high-throughput light-sheet tomography platform (HLTP), which adopts inverted setup light sheet imaging [150], deep learning-based fMOST, which uses a U-net convolutional neural network for real-time optical sectioning [84, 151], and cryo-MOST [91] and cryo-fMOST [92], for label-free frozen state imaging.

In general, the MOST series of technologies combines whole-brain sample embedding, automatic precision sectioning, and microscopic optical imaging, providing high-resolution optical imaging that traverses every voxel at the whole-brain scale. The collected datasets have excellent resolution, data quality, and integrity and can be used for quantitative analysis of the complete single-neuron anatomy, soma distribution, vascular networks, and other anatomical structures with colocalized cytoarchitecture information.

## Whole-brain Optical Imaging for Non-human Primates

The volume of the marmoset brain is ~16 times that of the mouse brain, so imaging of the entire marmoset brain can be achieved with a larger voxel size or increased imaging time [69, 136]. However, the volume of the macaque brain is >200 times that of the mouse brain, further requiring comprehensive improvements in tissue clearing, embedding, viral tracers, imaging, and image informatics for whole-brain profiling.

In 2019, Wang *et al*. proposed an inverted setup light-sheet microscope called volumetric imaging with synchronized on-the-fly-scan and readout (VISoR), which adopts a dynamic light sheet generated by scanning a Gaussian laser beam [152]. Moreover, a pipeline was developed for sample sectioning, clearing, imaging, and 3D image reconstruction. Finally, a complete volumetric dataset of ~50 300-μm slices of a whole mouse brain with a voxel size of ~0.5 μm × 0.5 μm × 3.5 μm was completed in <1.5 h. In 2021, Xu *et al*. combined the improved VISoR2 system with primate-optimized tissue sectioning and clearing, forming a pipeline capable of effective connectome-scale mapping in the large macaque brain [153]. A rhesus macaque brain was cut into ~250 consecutive 300-μm slices and cleared using this pipeline, and finally, all slices were imaged in 100 h with a voxel size of 1 μm × 1 μm × 2.5 μm. However, this 3D reconstruction of slice imaging data is inevitably hindered by data loss, nonlinear deformation, and interslice registration. In 2020, Luo *et al*. modified the resin-embedding process to embed large-volume tissues while preserving fluorescence. Using SI-fMOST for imaging, the complete cytoarchitectonic information of a macaque brain hemisphere and an intact ferret brain embedded with this method were obtained with submicron lateral resolution and an axial interval of 50 μm [154]. However, resin-embedded large-volume tissue samples are too rigid to be quickly and precisely sectioned, and thus, it is difficult to combine this embedding method with block-face imaging to achieve continuous high-resolution imaging. In 2022, Zhou *et al*. developed a novel poly-N-acryloyl glycinamide (PNAGA)-based embedding method that is suitable for intact macaque brains and can preserve the structure and fluorescence for extended periods, allowing rapid vibrating sectioning without serious deterioration of slice quality. By combining this method with a line-scan confocal imaging system, the cytoarchitectural information of a whole rhesus macaque brain was acquired in 80 days with a voxel size of 0.32 μm × 0.32 μm × 10 μm. Moreover, the projection pattern of the frontal cortex throughout the whole rhesus macaque brain hemisphere was obtained in 37 days with a voxel size of 0.65 μm × 0.65 μm × 3 μm [155]. Overall, whole-brain optical imaging techniques for nonhuman primates must be developed further to achieve finer and faster data acquisition.

## Large-scale Mesoscopic Whole-brain Imaging with Data Processing and Analysis

**Fig. 5:**
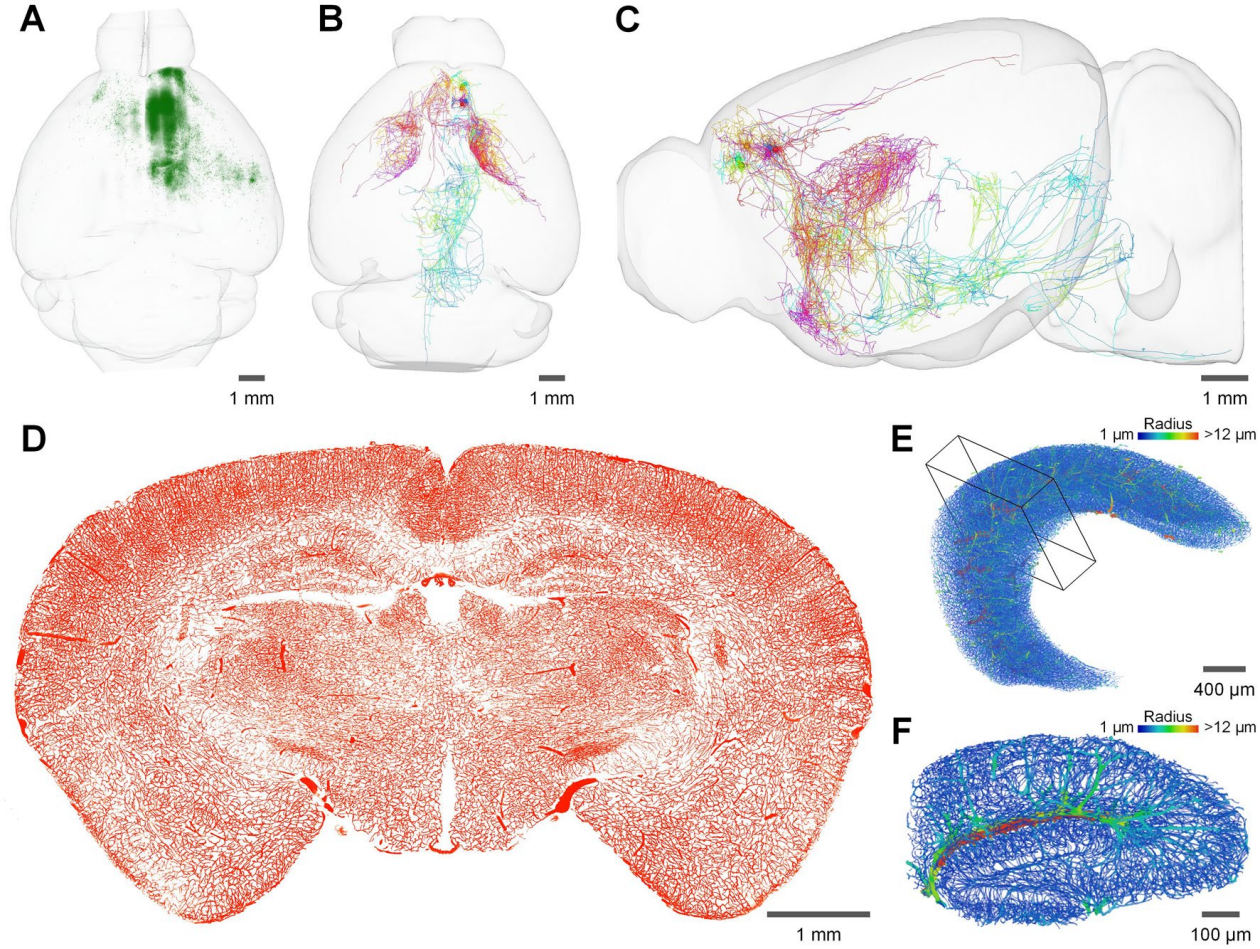
Presentation of different types of whole-brain imaging data. **A** Horizontal view of 3D rendering of whole-brain monosynaptic input neurons to secondary motor cortex (MOs)^PlxnD1+^ neurons. For retrograde monosynaptic labeling, adeno-associated helper virus (AAV helper) and modified rabies virus expressing GFP were injected into the MOs in the PlxnD1-2A-CreER mouse. This dataset was acquired by SI-fMOST. **B, C** Horizontal and sagittal views of the morphological reconstructions of 6 AAV-GFP-labeled neurons in the prelimbic area, showing the extent of axon projections across the brain and the variability of ipsilateral and contralateral projections. This dataset was acquired by HD-fMOST. **D** Minimum intensity projections of 100-μm thick coronal images of the whole-brain vasculature. The image pixel values are inverted. The mouse brain vascular system was labeled by intravenous injection of lectin-DyLight 488 followed by transcardiac perfusion of FITC-fluorescein gelatin solution and imaged by HD-fMOST. **E** 3D visualization of segmentation results of vessels in the hippocampus. **F** 3D visualization of the local 400-μm thick section of the hippocampus, as shown in the box in **F**. The vessels are color-coded by diameters for clarity in **E** and **F**.

The development of whole-brain optical imaging has resulted in an unprecedented amount of large-scale anatomical data, contributing to a method-driven renaissance in neuroanatomy [156]. These data enable brain-wide quantitative profiling of cells, circuits, and brain vascular structures. Figure 5 shows several typical whole-brain optical imaging results. Brain-wide cell analyses involve mapping the distributions of genetically defined cell types [73, 74, 134, 157], cell molecular features [58, 75, 158], and cells expressing immediate-early genes (IEGs) [132, 133, 158]. These datasets allow the precise dissection of the cellular composition of different brain regions and an understanding of the principles of mammalian brain organization [159]. This type of cell profiling application requires a relatively coarse resolution, so light-sheet microscopy is more widely used due to its high speed and the variety of available sample clearing and labeling methods. STP can also be used to obtain a high-quality whole-brain interval sampling dataset in several hours for cell analyses, but the 3D continuity is lost. Brain-wide mesoscopic connectivity data of neural circuits [135, 160–163] describe the long-range projections of specific neural populations in different brain regions, allowing an understanding of how information flows through neural circuits [164]. The more sophisticated brain-wide microscopic connectivity of single neurons is capable of showing the connectivity pathways of different brain regions at the cellular level, which is crucial for identifying cell types and defining how information is communicated between brain areas [131, 165–172]. This brain-wide observation of axons and dendritic fibers requires very high resolution and strict data quality uniformity, both of which are possible with block-face imaging methods. However, only the fMOST series of techniques and MouseLight have been realized on a large scale, especially for whole-brain, delicate single-neuron morphological tracing. In addition, whole-brain imaging can be used to reconstruct the brain-wide vascular network at the capillary level [140, 173, 174], providing morphological information for the study of the pathogenesis of vascular disease [175–177]. Table 2 compares the performance of these different types of whole-brain optical imaging methods and gives the recommended range of applications.

**Table 2:** Summary table of performance and recommended application range of different whole-brain optical imaging methods.

Whole-brain optical imaging		Sample preparation/	Typical voxel size	Data collection time	A suitable range of application	Whole-brain 3D dataset quality
Type	Typical method	Slicing method	(μm^3^)			
Mouse brain (light-sheet microscopy)	Ultramicroscopy 2007 [97]	Hydrophobic clearing	100–600 optical sections	6 h/mouse–8 h/mouse brain	Brain-wide cell analyses, mesoscopic connectivity, vascular network	★
	COLM 2014 [110]	Hydrogel-based clearing	/	~4 h or ~1.5 days/mouse brain (10× or 25× objective)	Brain-wide cell analyses, mesoscopic connectivity, vascular network Morphology of local neurons (axons, dendrites)	★★
	mesoSPIM 2019 [114]	Multiple clearing methods	1.6 × 1.6 × 2 6.55 × 6.55 × 5	13 min/mouse–16 min/mouse brain (1.6 μm × 1.6 μm × 2 μm)	Brain-wide cell analyses, mesoscopic connectivity, vascular network	★★
	ctASLM 2019 [115]	Multiple clearing methods	0.37 × 0.37 × 0.425 (overall) 0.167 × 0.167 × 0.2 (local)	5 frames/s–10 frames/s (up to 2048 × 2048 pixels)	Brain-wide cell analyses, mesoscopic connectivity, vascular network Fine morphology of local neurons (axons, dendrites, spines)	★★★
	Tiling LSM 2020 [118]	Multiple clearing methods	2.6 × 2.6 × 6 (overall) 0.3 × 0.3 × 1 (local)	5 h/mouse brain (2.6 μm × 2.6 μm × 6 μm)	Brain-wide cell analyses, mesoscopic connectivity, vascular network Morphology of local neurons (axons, dendrites)	★★★
	Hybrid OTLS 2022 [120]	Multiple clearing methods	0.45 × 0.46 × 2.91 (resolution) 4.41 × 4.09 × 5.48 (resolution)	~1 h/mouse brain (isotropic ~2 μm × 2 μm× 2 μm voxels)	Brain-wide cell analyses, mesoscopic connectivity, vascular network Morphology of local neurons (axons, dendrites)	★★★
	Mars-SPIM 2020 [125]	uDISCO	Isotropic ~1 (reconstructed)	~30 min/mouse brain	Brain-wide cell analyses, mesoscopic connectivity, vascular network	★★
	CACS LSM 2021 [126]	Hydrophobic clearing	Isotropic ~0.5 (reconstructed)	~10 min/mouse brain	Brain-wide cell analyses, mesoscopic connectivity, vascular network Morphology of local neurons (axons, dendrites)	★★
Mouse brain (block-face based)	STP 2012 [88]	Agarose-embedded Vibrating sectioning	0.5 × 0.5 × 50	24 h/mouse brain	Brain-wide cell analyses, mesoscopic connectivity	★★★
	MouseLight 2016 [130]	Dimethyl sulfoxide-clearing Vibrating sectioning	0.3 × 0.3 × 1	8 days/mouse–10 days/mouse brain	Brain-wide cell analyses, microscopic connectivity of single neurons, capillary network	★★★★
	FAST 2017 [136]	Agarose-embedded Vibrating sectioning	0.7 × 0.7 × 5	2.4 h/mouse–10 h/mouse brain	Brain-wide cell analyses, mesoscopic connectivity, vascular network	★★★
	SMART 2021 [138]	CUBIC-1 Vibrating sectioning	0.7 × 0.7 × 30 (overall) 0.3 × 0.3 × 1 (local)	20 h/part of mouse brain (0.3 μm × 0.3 μm × 1 μm)	Brain-wide cell analyses, mesoscopic connectivity, vascular network Morphology of local neurons (axons, dendrites)	★★
	MOST 2010 [86]	Nissl/Golgi staining Resin-embedded sectioning	0.33 × 0.33 × 1	242 h/mouse brain	Brain-wide Nissl stained cell analyses, capillary network Golgi stained neuron	★★
	fMOST 2013 [142]	Resin-embedded sectioning	1.0 × 0.8 × 1.0	447 h/mouse brain	Brain-wide cell analyses, microscopic connectivity of single neurons, capillary network	★★
	SI-fMOST 2016 [145]	Resin-embedded sectioning	0.32 × 0.32 × 2	3 days/mouse brain	Brain-wide cell analyses, microscopic connectivity of single neurons, capillary network	★★★★
	CS-fMOST 2021 [147]	Resin-embedded sectioning	0.23 × 0.23 × 1	6 days/mouse brain	Brain-wide cell analyses, microscopic connectivity of single neurons, capillary network Complete fine morphology of neurons (axons, dendrites, spines)	★★★★
	HD-fMOST 2021 [85]	Resin-embedded sectioning	0.32 × 0.32 × 1	111 h/mouse brain	Brain-wide cell analyses, microscopic connectivity of single neurons, capillary network Complete fine morphology of neurons (axons, dendrites, spines)	★★★★★
Nonhuman primates brain	VISoR2 2021 [153]	PuClear	1 × 1 × 2.5	94 h (macaque brain slices)	Brain-wide cell analyses, mesoscopic connectivity, vascular network	★
	Whole-brain imaging system for macaque brain 2022 [155]	PNAGA-embedded Vibrating sectioning	0.65 × 0.65 × 3	70 days/whole macaque brain	Brain-wide cell analyses, mesoscopic connectivity, vascular network	★★★★

The quantity and complexity of the data generated by the above studies preclude manual analysis, and extracting knowledge from these terabyte- and petabyte-scale data is likely a bottleneck that requires robust biological image analysis solutions for fully exploring the wealth of information [178–181]. The first step in parsing such image data is to preprocess the large amounts of raw data, consisting of many subvolumes of a single FOV. This step generally includes denoising, illumination correction, tiling, and quality control. Another frequently required preprocessing step is registration, where the images are accurately aligned to a standard reference atlas to allow comparison, fusion, and joint analysis between datasets from different samples. The next steps are to detect, segment, and track the target structure in the data, such as the soma or the entire neuron. Segmentation is arguably the most challenging step in biological image analysis, involving the detection of the presence of various structures and the grouping of pixels into targets of interest or backgrounds. Once segmentation and tracking are completed, further quantitative calculations can be performed to recognize the characteristics and patterns in the dataset and draw statistical conclusions. Manual involvement is often required in the above steps to inspect, correct, and annotate the results, which requires visualization of various high-dimensional images. Complete image analysis solutions require not only various powerful image analysis algorithms, data management tools, and computational tools [52, 182–188] but also powerful computing clusters for storing, computing and sharing large amounts of data. In short, hardware, software, and numerous processing steps are all required to form a systematic data analysis framework and pipeline.

## Prospects

In summary, we have provided a general overview of the technological pathways and evolution of whole-brain optical imaging, which has become an essential set of tools in neuroscience for resolving the anatomy of the brain at the mesoscopic level. However, whole-brain imaging of nonhuman primates remains a considerable challenge. Since the largest cross-section of the macaque brain has an area of >40 cm^2^, intact tissue clearing is challenging. In addition, standard light-sheet microscopy based on orthogonal illumination-detection optics is unable to reach such large lateral dimensions [113], and the working distance of the available objectives limits the axial imaging depth. Moreover, the large brain volume poses a severe challenge for the continuous and stable sectioning of large cross-sections over long periods. Therefore, whole-brain imaging of the macaque brain requires a systematic integration of chemical, mechanical, imaging, and computational tools to form a practical technology platform. Combining high-throughput block-face imaging methods such as line scanning or inverted setup light-sheet microscopy with tissue transformation [189] and physical sectioning may be a promising strategy.

Ultimately, however, the maximum throughput of all imaging techniques is limited by the properties of the camera. Using the latest ~1.1 gigapixel/s sCMOS camera, at least ~257 h are needed to acquire the data of a macaque brain with a voxel size of 0.3 μm × 0.3 μm × 1 μm, regardless of the movement and cutting times. Larger throughput cameras and higher numerical aperture objectives with a larger FOV will essentially increase the speed of whole-brain imaging. In addition, existing solutions for biological-image analysis are complex and rely more on computing clusters than individual workstations. The solutions also require researchers to identify the necessary processing steps, select the appropriate computing algorithms for each step, and tune their parameters. In the future, some data processing and analysis can be moved into the data acquisition process to enable intelligent imaging, reducing the amount of invalid data from the source of data generation and speeding up the imaging and data analysis processes. Moreover, with further developments in artificial intelligence technology and cloud services, researchers will be able to use their personal computers locally to automatically process the data. In addition, to facilitate the sharing and reusing of data, there is a need to develop relevant standardization and protocols to deal with the large amounts of data obtained by various whole-brain optical imaging methods, similar to medical imaging studies such as MRI [190, 191].

The seamless integration of labeling, whole-brain optical imaging, and informatics tools has begun to yield statistically robust conclusions and transform our understanding of the structural organization of the neural circuits in the mouse. With improvements in hardware performance and the advent of hybrid methods, the rapid and detailed analysis of the whole brain of nonhuman primates is within reach. The continued development of whole-brain optical imaging methods will further expand the possibilities for dissecting the neuronal network, providing critical information for deciphering structure-function relationships and understanding complex brain functions and human brain disorders.

## References

[1] Biswal BB, Mennes M, Zuo XN, Gohel S, Kelly C, Smith SM (2010). Toward discovery science of human brain function. Proc Natl Acad Sci U S A.

[2] Bassett DS, Gazzaniga MS (2011). Understanding complexity in the human brain. Trends Cogn Sci.

[3] Bargmann CI, Marder E (2013). From the connectome to brain function. Nat Methods.

[4] Sporns O, Bullmore ET (2014). From connections to function: The mouse brain connectome atlas. Cell.

[5] Lafarga M, Casafont I, Bengoechea R, Tapia O, Berciano MT (2009). Cajal’s contribution to the knowledge of the neuronal cell nucleus. Chromosoma.

[6] Mueller T, Kanis-Seyfried U (2019). On the life and work of Korbinian Brodmann (1868–1918). J Hist Neurosci.

[7] Amunts K, Mohlberg H, Bludau S, Zilles K (2020). Julich-Brain: A 3D probabilistic atlas of the human brain’s cytoarchitecture. Science.

[8] Paquola C, Royer J, Lewis LB, Lepage C, Glatard T, Wagstyl K (2021). The BigBrainWarp toolbox for integration of BigBrain 3D histology with multimodal neuroimaging. Elife.

[9] Glover P, Bowtell R (2009). MRI rides the wave. Nature.

[10] Amunts K, Zilles K, Zilles K (2015). Architectonic mapping of the human brain beyond Brodmann. Neuron.

[11] Shi Y, Toga AW (2017). Connectome imaging for mapping human brain pathways. Mol Psychiatry.

[12] Toga AW, Clark KA, Thompson PM, Shattuck DW, SDarrell Van Horn J (2012). Mapping the human connectome. Neurosurgery.

[13] Marx V (2013). Brain mapping in high resolution. Nature.

[14] Svara F, Förster D, Kubo F, Januszewski M, Dal Maschio M, Schubert PJ (2022). Automated synapse-level reconstruction of neural circuits in the larval zebrafish brain. Nat Methods.

[15] Turner NL, Macrina T, Bae JA, Yang R, Wilson AM, Schneider-Mizell C (2022). Reconstruction of neocortex: Organelles, compartments, cells, circuits, and activity. Cell.

[16] Livet J, Weissman TA, Kang H, Draft RW, Lu J, Bennis RA (2007). Transgenic strategies for combinatorial expression of fluorescent proteins in the nervous system. Nature.

[17] Taniguchi H, He M, Wu P, Kim S, Paik R, Sugino K (2011). A resource of Cre driver lines for genetic targeting of GABAergic neurons in cerebral cortex. Neuron.

[18] Susaki EA, Ueda HR (2016). Whole-body and whole-organ clearing and imaging techniques with single-cell resolution: Toward organism-level systems biology in mammals. Cell Chem Biol.

[19] Liu Q, Wu Y, Wang H, Jia F, Xu F (2022). Viral tools for neural circuit tracing. Neurosci Bull.

[20] Yang H, Xiong F, Song YG, Jiang HF, Qin HB, Zhou J (2021). HSV-1 H129-derived anterograde neural circuit tracers: Improvements, production, and applications. Neurosci Bull.

[21] Lichtman JW, Denk W (2011). The big and the small: Challenges of imaging the brain’s circuits. Science.

[22] Herculano-Houzel S, Mota B, Lent R (2006). Cellular scaling rules for rodent brains. Proc Natl Acad Sci U S A.

[23] Herculano-Houzel S (2009). The human brain in numbers: A linearly scaled-up primate brain. Front Hum Neurosci.

[24] Weisenburger S, Vaziri A (2018). A guide to emerging technologies for large-scale and whole-brain optical imaging of neuronal activity. Annu Rev Neurosci.

[25] Stuart G, Spruston N, Häusser M. Dendrite structure. In: Dendrites. 3rd ed. Oxford: Oxford University Press, 2016: 1–34.

[26] Müller B, Lang S, Dominietto M, Rudin M, Schulz G, Deyhle H, *et al*. High-resolution tomographic imaging of microvessels. In: Developments in X-ray Tomography VI. SPIE, 2008, 7078: 89–98.

[27] Osten P, Margrie TW (2013). Mapping brain circuitry with a light microscope. Nat Methods.

[28] Weisenburger S, Sandoghdar V (2015). Light microscopy: An ongoing contemporary revolution. Contemp Phys.

[29] Cole RW, Jinadasa T, Brown CM (2011). Measuring and interpreting point spread functions to determine confocal microscope resolution and ensure quality control. Nat Protoc.

[30] Shannon CE (1949). Communication in the presence of noise. Proceedings of the IRE.

[31] Kovacević N, Henderson JT, Chan E, Lifshitz N, Bishop J, Evans AC (2005). A three-dimensional MRI atlas of the mouse brain with estimates of the average and variability. Cereb Cortex.

[32] Seidlitz J, Sponheim C, Glen D, Ye FQ, Saleem KS, Leopold DA (2018). A population MRI brain template and analysis tools for the macaque. Neuroimage.

[33] Capitanio JP, Emborg ME (2008). Contributions of non-human primates to neuroscience research. Lancet.

[34] Roelfsema PR, Treue S (2014). Basic neuroscience research with nonhuman primates: A small but indispensable component of biomedical research. Neuron.

[35] Miller CT, Freiwald WA, Leopold DA, Mitchell JF, Silva AC, Wang X (2016). Marmosets: A neuroscientific model of human social behavior. Neuron.

[36] Kennedy H, Dehay C (2020). From mouse to man—a bridge too far?. Natl Sci Rev.

[37] Huang ZJ, Luo L (2015). It takes the world to understand the brain. Science.

[38] Grillner S, Ip N, Koch C, Koroshetz W, Okano H, Polachek M (2016). Worldwide initiatives to advance brain research. Nat Neurosci.

[39] Feng G, Jensen FE, Greely HT, Okano H, Treue S, Roberts AC (2020). Opportunities and limitations of genetically modified nonhuman primate models for neuroscience research. Proc Natl Acad Sci U S A.

[40] Okano H, Miyawaki A, Kasai K (2015). Brain/MINDS: Brain-mapping Project in Japan. Philos Trans R Soc Lond B Biol Sci.

[41] Poo MM, Du JL, Ip NY, Xiong ZQ, Xu B, Tan T (2016). China brain project: Basic neuroscience, brain diseases, and brain-inspired computing. Neuron.

[42] Herculano-Houzel S, Collins CE, Wong P, Kaas JH (2007). Cellular scaling rules for primate brains. Proc Natl Acad Sci U S A.

[43] Helmchen F, Denk W (2005). Deep tissue two-photon microscopy. Nat Methods.

[44] Yoon S, Kim M, Jang M, Choi Y, Choi W, Kang S (2020). Deep optical imaging within complex scattering media. Nat Rev Phys.

[45] Conchello JA, Lichtman JW (2005). Optical sectioning microscopy. Nat Methods.

[46] Mertz J (2011). Optical sectioning microscopy with planar or structured illumination. Nat Methods.

[47] Wu Y, Shroff H (2018). Faster, sharper, and deeper: Structured illumination microscopy for biological imaging. Nat Methods.

[48] Stelzer EHK, Strobl F, Chang BJ, Preusser F, Preibisch S, McDole K (2021). Light sheet fluorescence microscopy. Nat Rev Methods Primers.

[49] Spalteholz W. Über das durchsichtigmachen von menschlichen und tierischen Präparaten S. Hierzel, Leipzig, 1914.

[50] Richardson DS, Lichtman JW (2015). Clarifying tissue clearing. Cell.

[51] Richardson DS, Guan W, Matsumoto K, Pan C, Chung K, Ertürk A (2021). Tissue clearing. Nat Rev Methods Primers.

[52] Ueda HR, Ertürk A, Chung K, Gradinaru V, Chédotal A, Tomancak P (2020). Tissue clearing and its applications in neuroscience. Nat Rev Neurosci.

[53] Tainaka K, Kuno A, Kubota SI, Murakami T, Ueda HR (2016). Chemical principles in tissue clearing and staining protocols for whole-body cell profiling. Annu Rev Cell Dev Biol.

[54] Ertürk A, Becker K, Jährling N, Mauch CP, Hojer CD, Egen JG (2012). Three-dimensional imaging of solvent-cleared organs using 3DISCO. Nat Protoc.

[55] Renier N, Wu Z, Simon DJ, Yang J, Ariel P, Tessier-Lavigne M (2014). iDISCO: A simple, rapid method to immunolabel large tissue samples for volume imaging. Cell.

[56] Pan C, Cai R, Quacquarelli FP, Ghasemigharagoz A, Lourbopoulos A, Matryba P (2016). Shrinkage-mediated imaging of entire organs and organisms using uDISCO. Nat Methods.

[57] Qi Y, Yu T, Xu J, Wan P, Ma Y, Zhu J, *et al*. FDISCO: Advanced solvent-based clearing method for imaging whole organs. Sci Adv 2019, 5: eaau8355. 10.1126/sciadv.aau8355 PMC6357753 30746463

[58] Cai R, Pan C, Ghasemigharagoz A, Todorov MI, Förstera B, Zhao S (2019). Panoptic imaging of transparent mice reveals whole-body neuronal projections and skull-meninges connections. Nat Neurosci.

[59] Hahn C, Becker K, Saghafi S, Pende M, Avdibašić A, Foroughipour M (2019). High-resolution imaging of fluorescent whole mouse brains using stabilised organic media (sDISCO). J Biophotonics.

[60] Jing D, Zhang S, Luo W, Gao X, Men Y, Ma C (2018). Tissue clearing of both hard and soft tissue organs with the PEGASOS method. Cell Res.

[61] Liu YC, Chiang AS (2003). High-resolution confocal imaging and three-dimensional rendering. Methods.

[62] Hama H, Kurokawa H, Kawano H, Ando R, Shimogori T, Noda H (2011). Scale: A chemical approach for fluorescence imaging and reconstruction of transparent mouse brain. Nat Neurosci.

[63] Hama H, Hioki H, Namiki K, Hoshida T, Kurokawa H, Ishidate F (2015). ScaleS: An optical clearing palette for biological imaging. Nat Neurosci.

[64] Ke MT, Fujimoto S, Imai T (2013). SeeDB: A simple and morphology-preserving optical clearing agent for neuronal circuit reconstruction. Nat Neurosci.

[65] Ke MT, Nakai Y, Fujimoto S, Takayama R, Yoshida S, Kitajima TS (2016). Super-resolution mapping of neuronal circuitry with an index-optimized clearing agent. Cell Rep.

[66] Yu T, Zhu J, Li Y, Ma Y, Wang J, Cheng X (1964). RTF: A rapid and versatile tissue optical clearing method. Sci Rep.

[67] Hou B, Zhang D, Zhao S, Wei M, Yang Z, Wang S (2015). Scalable and DiI-compatible optical clearance of the mammalian brain. Front Neuroanat.

[68] Chen L, Li G, Li Y, Li Y, Zhu H, Tang L (2017). UbasM: An effective balanced optical clearing method for intact biomedical imaging. Sci Rep.

[69] Susaki EA, Tainaka K, Perrin D, Kishino F, Tawara T, Watanabe TM (2014). Whole-brain imaging with single-cell resolution using chemical cocktails and computational analysis. Cell.

[70] Susaki EA, Tainaka K, Perrin D, Yukinaga H, Kuno A, Ueda HR (2015). Advanced CUBIC protocols for whole-brain and whole-body clearing and imaging. Nat Protoc.

[71] Kubota SI, Takahashi K, Nishida J, Morishita Y, Ehata S, Tainaka K (2017). Whole-body profiling of cancer metastasis with single-cell resolution. Cell Rep.

[72] Tainaka K, Murakami TC, Susaki EA, Shimizu C, Saito R, Takahashi K (2018). Chemical landscape for tissue clearing based on hydrophilic reagents. Cell Rep.

[73] Murakami TC, Mano T, Saikawa S, Horiguchi SA, Shigeta D, Baba K (2018). A three-dimensional single-cell-resolution whole-brain atlas using CUBIC-X expansion microscopy and tissue clearing. Nat Neurosci.

[74] Matsumoto K, Mitani TT, Horiguchi SA, Kaneshiro J, Murakami TC, Mano T (2019). Advanced CUBIC tissue clearing for whole-organ cell profiling. Nat Protoc.

[75] Chung K, Wallace J, Kim SY, Kalyanasundaram S, Andalman AS, Davidson TJ (2013). Structural and molecular interrogation of intact biological systems. Nature.

[76] Yang B, Treweek JB, Kulkarni RP, Deverman BE, Chen CK, Lubeck E (2014). Single-cell phenotyping within transparent intact tissue through whole-body clearing. Cell.

[77] Treweek JB, Chan KY, Flytzanis NC, Yang B, Deverman BE, Greenbaum A (2015). Whole-body tissue stabilization and selective extractions via tissue-hydrogel hybrids for high-resolution intact circuit mapping and phenotyping. Nat Protoc.

[78] Murray E, Cho JH, Goodwin D, Ku T, Swaney J, Kim SY (2015). Simple, scalable proteomic imaging for high-dimensional profiling of intact systems. Cell.

[79] Park YG, Sohn CH, Chen R, McCue M, Yun DH, Drummond GT (2019). Protection of tissue physicochemical properties using polyfunctional crosslinkers. Nat Biotechnol.

[80] Ku T, Guan W, Evans NB, Sohn CH, Albanese A, Kim JG (2020). Elasticizing tissues for reversible shape transformation and accelerated molecular labeling. Nat Methods.

[81] Hillman EMC, Voleti V, Li W, Yu H (2019). Light-sheet microscopy in neuroscience. Annu Rev Neurosci.

[82] Diel EE, Lichtman JW, Richardson DS (2020). Tutorial: Avoiding and correcting sample-induced spherical aberration artifacts in 3D fluorescence microscopy. Nat Protoc.

[83] Xiong H, Zhou Z, Zhu M, Lv X, Li A, Li S (2014). Chemical reactivation of quenched fluorescent protein molecules enables resin-embedded fluorescence microimaging. Nat Commun.

[84] Zhang X, Chen Y, Ning K, Zhou C, Han Y, Gong H (2018). Deep learning optical-sectioning method. Opt Express.

[85] Zhong Q, Li A, Jin R, Zhang D, Li X, Jia X (2021). High-definition imaging using line-illumination modulation microscopy. Nat Methods.

[86] Li A, Gong H, Zhang B, Wang Q, Yan C, Wu J (2010). Micro-optical sectioning tomography to obtain a high-resolution atlas of the mouse brain. Science.

[87] Wang Q, Li A, Gong H, Xu D, Luo Q (2012). Quantitative study on the hygroscopic expansion of spurr resin to obtain a high-resolution atlas of the mouse brain. Exp Biol Med (Maywood).

[88] Ragan T, Kadiri LR, Venkataraju KU, Bahlmann K, Sutin J, Taranda J (2012). Serial two-photon tomography for automated *ex vivo* mouse brain imaging. Nat Methods.

[89] Jiang T, Long B, Gong H, Xu T, Li X, Duan Z (2017). A platform for efficient identification of molecular phenotypes of brain-wide neural circuits. Sci Rep.

[90] Li Y, Ding Z, Deng L, Fan G, Zhang Q, Gong H, *et al*. Precision vibratome for high-speed ultrathin biotissue cutting and organ-wide imaging. iScience 2021, 24: 103016. 10.1016/j.isci.2021.103016 PMC8426277 34522859

[91] Luo Y, Wang A, Liu M, Lei T, Zhang X, Gao Z (2017). Label-free brainwide visualization of senile plaque using cryo-micro-optical sectioning tomography. Opt Lett.

[92] Deng L, Chen J, Li Y, Han Y, Fan G, Yang J, *et al*. Cryo-fluorescence micro-optical sectioning tomography for volumetric imaging of various whole organs with subcellular resolution. iScience 2022, 25: 104805. 10.1016/j.isci.2022.104805 PMC9389242 35992061

[93] Zhanmu O, Zhao P, Yang Y, Yang X, Gong H, Li X (2019). Maintenance of fluorescence during paraffin embedding of fluorescent protein-labeled specimens. Front Neurosci.

[94] Zhanmu O, Yang X, Gong H, Li X (2020). Paraffin-embedding for large volume bio-tissue. Sci Rep.

[95] Siedentopf H, Zsigmondy R (1902). Uber sichtbarmachung und größenbestimmung ultramikoskopischer teilchen, mit besonderer anwendung auf goldrubingläser. Annalen der Physik.

[96] Huisken J, Swoger J, Del Bene F, Wittbrodt J, Stelzer EH (2004). Optical sectioning deep inside live embryos by selective plane illumination microscopy. Science.

[97] Dodt HU, Leischner U, Schierloh A, Jährling N, Mauch CP, Deininger K (2007). Ultramicroscopy: Three-dimensional visualization of neuronal networks in the whole mouse brain. Nat Methods.

[98] Keller PJ, Ahrens MB (2015). Visualizing whole-brain activity and development at the single-cell level using light-sheet microscopy. Neuron.

[99] Ueda HR, Dodt HU, Osten P, Economo MN, Chandrashekar J, Keller PJ (2020). Whole-brain profiling of cells and circuits in mammals by tissue clearing and light-sheet microscopy. Neuron.

[100] Keller PJ, Schmidt AD, Wittbrodt J, Stelzer EH (2008). Reconstruction of zebrafish early embryonic development by scanned light sheet microscopy. Science.

[101] Fahrbach FO, Rohrbach A (2012). Propagation stability of self-reconstructing Bessel beams enables contrast-enhanced imaging in thick media. Nat Commun.

[102] Planchon TA, Gao L, Milkie DE, Davidson MW, Galbraith JA, Galbraith CG (2011). Rapid three-dimensional isotropic imaging of living cells using Bessel beam plane illumination. Nat Methods.

[103] Vettenburg T, Dalgarno HIC, Nylk J, Coll-Lladó C, Ferrier DEK, Čižmár T (2014). Light-sheet microscopy using an Airy beam. Nat Methods.

[104] Legant WR, Shao L, Grimm JB, Brown TA, Milkie DE, Avants BB (2016). High-density three-dimensional localization microscopy across large volumes. Nat Methods.

[105] Dunsby C (2008). Optically sectioned imaging by oblique plane microscopy. Opt Express.

[106] Saghafi S, Becker K, Hahn C, Dodt HU (2014). 3D-ultramicroscopy utilizing aspheric optics. J Biophotonics.

[107] Mertz J, Kim J (2010). Scanning light-sheet microscopy in the whole mouse brain with HiLo background rejection. J Biomed Opt.

[108] Silvestri L, Bria A, Sacconi L, Iannello G, Pavone FS (2012). Confocal light sheet microscopy: Micron-scale neuroanatomy of the entire mouse brain. Opt Express.

[109] Baumgart E, Kubitscheck U (2012). Scanned light sheet microscopy with confocal slit detection. Opt Express.

[110] Tomer R, Ye L, Hsueh B, Deisseroth K (2014). Advanced CLARITY for rapid and high-resolution imaging of intact tissues. Nat Protoc.

[111] Tomer R, Lovett-Barron M, Kauvar I, Andalman A, Burns VM, Sankaran S (2015). SPED light sheet microscopy: Fast mapping of biological system structure and function. Cell.

[112] Dean KM, Roudot P, Welf ES, Danuser G, Fiolka R (2015). Deconvolution-free subcellular imaging with axially swept light sheet microscopy. Biophys J.

[113] Migliori B, Datta MS, Dupre C, Apak MC, Asano S, Gao R (2018). Light sheet theta microscopy for rapid high-resolution imaging of large biological samples. BMC Biol.

[114] Voigt FF, Kirschenbaum D, Platonova E, Pagès S, Campbell RAA, Kastli R (2019). The mesoSPIM initiative: Open-source light-sheet microscopes for imaging cleared tissue. Nat Methods.

[115] Chakraborty T, Driscoll MK, Jeffery E, Murphy MM, Roudot P, Chang BJ (2019). Light-sheet microscopy of cleared tissues with isotropic, subcellular resolution. Nat Methods.

[116] Zhang Z, Yao X, Yin X, Ding Z, Huang T, Huo Y (2021). Multi-scale light-sheet fluorescence microscopy for fast whole brain imaging. Front Neuroanat.

[117] Gao L (2015). Extend the field of view of selective plan illumination microscopy by tiling the excitation light sheet. Opt Express.

[118] Chen Y, Li X, Zhang D, Wang C, Feng R, Li X (2020). A versatile tiling light sheet microscope for imaging of cleared tissues. Cell Rep.

[119] Glaser AK, Reder NP, Chen Y, Yin C, Wei L, Kang S (2019). Multi-immersion open-top light-sheet microscope for high-throughput imaging of cleared tissues. Nat Commun.

[120] Glaser AK, Bishop KW, Barner LA, Susaki EA, Kubota SI, Gao G (2022). A hybrid open-top light-sheet microscope for versatile multi-scale imaging of cleared tissues. Nat Methods.

[121] Krzic U, Gunther S, Saunders TE, Streichan SJ, Hufnagel L (2012). Multiview light-sheet microscope for rapid in toto imaging. Nat Methods.

[122] Chhetri RK, Amat F, Wan Y, Höckendorf B, Lemon WC, Keller PJ (2015). Whole-animal functional and developmental imaging with isotropic spatial resolution. Nat Methods.

[123] Swoger J, Verveer P, Greger K, Huisken J, Stelzer EH (2007). Multi-view image fusion improves resolution in three-dimensional microscopy. Opt Express.

[124] Preibisch S, Amat F, Stamataki E, Sarov M, Singer RH, Myers E (2014). Efficient Bayesian-based multiview deconvolution. Nat Methods.

[125] Nie J, Liu S, Yu T, Li Y, Ping J, Wan P (2020). Fast, 3D isotropic imaging of whole mouse brain using multiangle-resolved subvoxel SPIM. Adv Sci (Weinh).

[126] Fang C, Yu T, Chu T, Feng W, Zhao F, Wang X (2021). Minutes-timescale 3D isotropic imaging of entire organs at subcellular resolution by content-aware compressed-sensing light-sheet microscopy. Nat Commun.

[127] Zingg B, Hintiryan H, Gou L, Song MY, Bay M, Bienkowski MS (2014). Neural networks of the mouse neocortex. Cell.

[128] Lin MK, Takahashi YS, Huo BX, Hanada M, Nagashima J, Hata J (2019). A high-throughput neurohistological pipeline for brain-wide mesoscale connectivity mapping of the common marmoset. Elife.

[129] Ragan T, Sylvan JD, Kim KH, Huang H, Bahlmann K, Lee RT (2007). High-resolution whole organ imaging using two-photon tissue cytometry. J Biomed Opt.

[130] Economo MN, Clack NG, Lavis LD, Gerfen CR, Svoboda K, Myers EW (2016). A platform for brain-wide imaging and reconstruction of individual neurons. Elife.

[131] Winnubst J, Bas E, Ferreira TA, Wu Z, Economo MN, Edson P (2019). Reconstruction of 1000 projection neurons reveals new cell types and organization of long-range connectivity in the mouse brain. Cell.

[132] Vousden DA, Epp J, Okuno H, Nieman BJ, Eede M, Jun D (2015). Whole-brain mapping of behaviourally induced neural activation in mice. Brain Struct Funct.

[133] Kim Y, Venkataraju KU, Pradhan K, Mende C, Taranda J, Turaga SC (2015). Mapping social behavior-induced brain activation at cellular resolution in the mouse. Cell Rep.

[134] Kim Y, Yang GR, Pradhan K, Venkataraju KU, Bota M, García Del Molino LC (2017). Brain-wide maps reveal stereotyped cell-type-based cortical architecture and subcortical sexual dimorphism. Cell.

[135] Oh SW, Harris JA, Ng L, Winslow B, Cain N, Mihalas S (2014). A mesoscale connectome of the mouse brain. Nature.

[136] Seiriki K, Kasai A, Hashimoto T, Schulze W, Niu M, Yamaguchi S (2017). High-speed and scalable whole-brain imaging in rodents and primates. Neuron.

[137] Seiriki K, Kasai A, Nakazawa T, Niu M, Naka Y, Tanuma M (2019). Whole-brain block-face serial microscopy tomography at subcellular resolution using FAST. Nat Protoc.

[138] Chen H, Huang T, Yang Y, Yao X, Huo Y, Wang Y (2021). Sparse imaging and reconstruction tomography for high-speed high-resolution whole-brain imaging. Cell Rep Methods.

[139] Zhang B, Li A, Yang Z, Wu J, Luo Q, Gong H (2011). Modified Golgi-Cox method for micrometer scale sectioning of the whole mouse brain. J Neurosci Methods.

[140] Wu J, He Y, Yang Z, Guo C, Luo Q, Zhou W (2014). 3D BrainCV: Simultaneous visualization and analysis of cells and capillaries in a whole mouse brain with one-micron voxel resolution. Neuroimage.

[141] Wu J, Guo C, Chen S, Jiang T, He Y, Ding W (2016). Direct 3D analyses reveal barrel-specific vascular distribution and cross-barrel branching in the mouse barrel cortex. Cereb Cortex.

[142] Gong H, Zeng S, Yan C, Lv X, Yang Z, Xu T (2013). Continuously tracing brain-wide long-distance axonal projections in mice at a one-micron voxel resolution. Neuroimage.

[143] Yang Z, Hu B, Zhang Y, Luo Q, Gong H (2013). Development of a plastic embedding method for large-volume and fluorescent-protein-expressing tissues. PLoS One.

[144] Xu D, Jiang T, Li A, Hu B, Feng Z, Gong H (2013). Fast optical sectioning obtained by structured illumination microscopy using a digital mirror device. J Biomed Opt.

[145] Gong H, Xu D, Yuan J, Li X, Guo C, Peng J (2016). High-throughput dual-colour precision imaging for brain-wide connectome with cytoarchitectonic landmarks at the cellular level. Nat Commun.

[146] Yang T, Zheng T, Shang Z, Wang X, Lv X, Yuan J (2015). Rapid imaging of large tissues using high-resolution stage-scanning microscopy. Biomed Opt Express.

[147] Wang X, Xiong H, Liu Y, Yang T, Li A, Huang F (2021). Chemical sectioning fluorescence tomography: High-throughput, high-contrast, multicolor, whole-brain imaging at subcellular resolution. Cell Rep.

[148] Zheng T, Yang Z, Li A, Lv X, Zhou Z, Wang X (2013). Visualization of brain circuits using two-photon fluorescence micro-optical sectioning tomography. Opt Express.

[149] Chen X, Zhang X, Zhong Q, Sun Q, Peng J, Gong H (2018). Simultaneous acquisition of neuronal morphology and cytoarchitecture in the same Golgi-stained brain. Biomed Opt Express.

[150] Yang X, Zhang Q, Huang F, Bai K, Guo Y, Zhang Y (2018). High-throughput light sheet tomography platform for automated fast imaging of whole mouse brain. J Biophotonics.

[151] Ning K, Zhang X, Gao X, Jiang T, Wang H, Chen S (2020). Deep-learning-based whole-brain imaging at single-neuron resolution. Biomed Opt Express.

[152] Wang H, Zhu Q, Ding L, Shen Y, Yang CY, Xu F (2019). Scalable volumetric imaging for ultrahigh-speed brain mapping at synaptic resolution. Natl Sci Rev.

[153] Xu F, Shen Y, Ding L, Yang CY, Tan H, Wang H (2021). High-throughput mapping of a whole *Rhesus* monkey brain at micrometer resolution. Nat Biotechnol.

[154] Luo T, Deng L, Li A, Zhou C, Shao S, Sun Q, *et al*. Scalable resin embedding method for large-volume brain tissues with high fluorescence preservation capacity. iScience 2020, 23: 101717. 10.1016/j.isci.2020.101717 PMC7645060 33196032

[155] Zhou C, Yang X, Wu S, Zhong Q, Luo T, Li A (2022). Continuous subcellular resolution three-dimensional imaging on intact macaque brain. Sci Bull (Beijing).

[156] BRAIN Initiative Cell Census Network (BICCN). A multimodal cell census and atlas of the mammalian primary motor cortex. Nature 2021, 598: 86–102. 10.1038/s41586-021-03950-0 PMC8494634 34616075

[157] Zhang C, Yan C, Ren M, Li A, Quan T, Gong H (2017). A platform for stereological quantitative analysis of the brain-wide distribution of type-specific neurons. Sci Rep.

[158] Renier N, Adams EL, Kirst C, Wu Z, Azevedo R, Kohl J (2016). Mapping of brain activity by automated volume analysis of immediate early genes. Cell.

[159] Wang Q, Ding SL, Li Y, Royall J, Feng D, Lesnar P (2020). The Allen mouse brain common coordinate framework: A 3D reference atlas. Cell.

[160] Matho KS, Huilgol D, Galbavy W, He M, Kim G, An X (2021). Genetic dissection of the glutamatergic neuron system in cerebral cortex. Nature.

[161] Xu Z, Feng Z, Zhao M, Sun Q, Deng L, Jia X (2021). Whole-brain connectivity atlas of glutamatergic and GABAergic neurons in the mouse dorsal and Median raphe nuclei. Elife.

[162] Yang Y, Jiang T, Jia X, Yuan J, Li X, Gong H (2022). Whole-brain connectome of GABAergic neurons in the mouse zona incerta. Neurosci Bull.

[163] Zhao M, Ren M, Jiang T, Jia X, Wang X, Li A (2022). Whole-brain direct inputs to and axonal projections from excitatory and inhibitory neurons in the mouse primary auditory area. Neurosci Bull.

[164] Mitra PP. The circuit architecture of whole brains at the mesoscopic scale. Neuron 2014, 83: 1273–1283. 10.1016/j.neuron.2014.08.055 PMC4256953 25233311

[165] Economo MN, Winnubst J, Bas E, Ferreira TA, Chandrashekar J (2019). Single-neuron axonal reconstruction: The search for a wiring diagram of the brain. J Comp Neurol.

[166] Peng H, Xie P, Liu L, Kuang X, Wang Y, Qu L (2021). Morphological diversity of single neurons in molecularly defined cell types. Nature.

[167] Foster NN, Barry J, Korobkova L, Garcia L, Gao L, Becerra M (2021). The mouse cortico-basal Ganglia-thalamic network. Nature.

[168] Tian J, Ren M, Zhao P, Luo S, Chen Y, Xu X (2022). Dissection of the long-range projections of specific neurons at the synaptic level in the whole mouse brain. Proc Natl Acad Sci U S A.

[169] Sun Q, Zhang J, Li A, Yao M, Liu G, Chen S (2022). Acetylcholine deficiency disrupts extratelencephalic projection neurons in the prefrontal cortex in a mouse model of Alzheimer’s disease. Nat Commun.

[170] Gao L, Liu S, Gou L, Hu Y, Liu Y, Deng L (2022). Single-neuron projectome of mouse prefrontal cortex. Nat Neurosci.

[171] Muñoz-Castañeda R, Zingg B, Matho KS, Chen X, Wang Q, Foster NN (2021). Cellular anatomy of the mouse primary motor cortex. Nature.

[172] Wang J, Sun P, Lv X, Jin S, Li A, Kuang J (2021). Divergent projection patterns revealed by reconstruction of individual neurons in orbitofrontal cortex. Neurosci Bull.

[173] di Giovanna AP, Tibo A, Silvestri L, Müllenbroich MC, Costantini I, Allegra Mascaro AL (2018). Whole-brain vasculature reconstruction at the single capillary level. Sci Rep.

[174] Xiong B, Li A, Lou Y, Chen S, Long B, Peng J (2017). Precise cerebral vascular atlas in stereotaxic coordinates of whole mouse brain. Front Neuroanat.

[175] Zhang X, Yin X, Zhang J, Li A, Gong H, Luo Q (2019). High-resolution mapping of brain vasculature and its impairment in the hippocampus of Alzheimer’s disease mice. Natl Sci Rev.

[176] Tang J, Zhu H, Tian X, Wang H, Liu S, Liu K (2022). Extension of endocardium-derived vessels generate coronary arteries in neonates. Circ Res.

[177] He XZ, Li X, Li ZH, Meng JC, Mao RT, Zhang XK (2022). High-resolution 3D demonstration of regional heterogeneity in the glymphatic system. J Cereb Blood Flow Metab.

[178] Frégnac Y (2017). Big data and the industrialization of neuroscience: A safe roadmap for understanding the brain?. Science.

[179] Amunts K, Lippert T (2021). Brain research challenges supercomputing. Science.

[180] Meijering E, Carpenter AE, Peng H, Hamprecht FA, Olivo-Marin JC (2016). Imagining the future of bioimage analysis. Nat Biotechnol.

[181] Li A, Guan Y, Gong H, Luo Q (2019). Challenges of processing and analyzing big data in mesoscopic whole-brain imaging. Genomics Proteomics Bioinformatics.

[182] Quan T, Zheng T, Yang Z, Ding W, Li S, Li J (2013). NeuroGPS: Automated localization of neurons for brain circuits using L1 minimization model. Sci Rep.

[183] Zhou H, Li S, Li A, Huang Q, Xiong F, Li N (2021). GTree: An open-source tool for dense reconstruction of brain-wide neuronal population. Neuroinformatics.

[184] Hörl D, Rojas Rusak F, Preusser F, Tillberg P, Randel N, Chhetri RK (2019). BigStitcher: Reconstructing high-resolution image datasets of cleared and expanded samples. Nat Methods.

[185] Cheeseman BL, Günther U, Gonciarz K, Susik M, Sbalzarini IF (2018). Adaptive particle representation of fluorescence microscopy images. Nat Commun.

[186] Saalfeld S, Cardona A, Hartenstein V, Tomancak P (2009). CATMAID: Collaborative annotation toolkit for massive amounts of image data. Bioinformatics.

[187] Qu L, Li Y, Xie P, Liu L, Wang Y, Wu J (2022). Cross-modal coherent registration of whole mouse brains. Nat Methods.

[188] Li Y, Liu X, Jia X, Jiang T, Wu J, Zhang Q, *et al*. A high-performance deep-learning-based pipeline for whole-brain vasculature segmentation at the capillary resolution. Bioinformatics 2023, 39: btad145. 10.1093/bioinformatics/btad145 PMC10068744 36946294

[189] Choi SW, Guan W, Chung K (2021). Basic principles of hydrogel-based tissue transformation technologies and their applications. Cell.

[190] Gorgolewski KJ, Auer T, Calhoun VD, Craddock RC, Das S, Duff EP (2016). The brain imaging data structure, a format for organizing and describing outputs of neuroimaging experiments. Sci Data.

[191] Mantri M, Taran S, Sunder G (2022). DICOM integration libraries for medical image interoperability: A technical review. IEEE Rev Biomed Eng.

